# Association of hypothyroidism with survival in pancreatic cancer: retrospective cohort study

**DOI:** 10.1093/bjsopen/zrad119

**Published:** 2024-01-09

**Authors:** Ingrid Garajová, Annalisa Comandatore, Lenka Boyd, Mahsoem Ali, Fabio Gelsomino, Stefania de Lorenzo, Giuseppe Pedrazzi, Andrea Spallanzani, Giulio Martinelli, Rita Balsano, Francesco Leonardi, Matteo Palmeri, Geert Kazemier, Gregorio Di Franco, Simone Guadagni, Niccolò Furbetta, Manuel Gentiluomo, Niccolò Ramacciotti, Giulio Di Candio, Elisa Giovannetti, Luca Morelli

**Affiliations:** Medical Oncology Unit, University Hospital of Parma, Parma, Italy; General Surgery Unit, Department of Translational Research and New Technologies in Medicine and Surgery, University of Pisa, Pisa, Italy; Department of Medical Oncology, Lab of Medical Oncology, Cancer Center Amsterdam, Amsterdam UMC, VU University Medical Center (VUmc), Amsterdam, The Netherlands; Department of Medical Oncology, Lab of Medical Oncology, Cancer Center Amsterdam, Amsterdam UMC, VU University Medical Center (VUmc), Amsterdam, The Netherlands; Department of Medical Oncology, Lab of Medical Oncology, Cancer Center Amsterdam, Amsterdam UMC, VU University Medical Center (VUmc), Amsterdam, The Netherlands; Department of Oncology and Hematology, University Hospital of Modena, Modena, Italy; Oncology Unit, Azienda USL Bologna, Bologna, Italy; Department of Medicine and Surgery, University of Parma, Parma, Italy; Department of Oncology and Hematology, University Hospital of Modena, Modena, Italy; Department of Oncology and Hematology, University Hospital of Modena, Modena, Italy; Medical Oncology Unit, University Hospital of Parma, Parma, Italy; Medical Oncology Unit, University Hospital of Parma, Parma, Italy; General Surgery Unit, Department of Translational Research and New Technologies in Medicine and Surgery, University of Pisa, Pisa, Italy; Department of Surgery, Amsterdam UMC, VU University Medical Center, Amsterdam, The Netherlands; General Surgery Unit, Department of Translational Research and New Technologies in Medicine and Surgery, University of Pisa, Pisa, Italy; General Surgery Unit, Department of Translational Research and New Technologies in Medicine and Surgery, University of Pisa, Pisa, Italy; General Surgery Unit, Department of Translational Research and New Technologies in Medicine and Surgery, University of Pisa, Pisa, Italy; Department of Biology, University of Pisa, Pisa, Italy; General Surgery Unit, Department of Translational Research and New Technologies in Medicine and Surgery, University of Pisa, Pisa, Italy; General Surgery Unit, Department of Translational Research and New Technologies in Medicine and Surgery, University of Pisa, Pisa, Italy; Department of Medical Oncology, Lab of Medical Oncology, Cancer Center Amsterdam, Amsterdam UMC, VU University Medical Center (VUmc), Amsterdam, The Netherlands; Cancer Pharmacology Lab, AIRC Start-Up Unit, Fondazione Pisana per la Scienza, San Giuliano Terme, PI, Pisa, Italy; General Surgery Unit, Department of Translational Research and New Technologies in Medicine and Surgery, University of Pisa, Pisa, Italy

## Introduction

Pancreatic ductal adenocarcinoma (PDAC) remains one of the most aggressive tumours, with a 5-year survival rate of approximately 8 per cent^1^. This is mostly due to the delay in early diagnosis and to tumour resistance against treatments, despite considerable recent effort in both preclinical and clinical research^2–5^. New studies are needed to identify potential risk factors of progression and incidence of PDAC.

Thyroid disorders are a common public health problem worldwide. The most common type of thyroid disorder is hypothyroidism^6^, which is characterized by insufficient levels of thyroxine (T4), along with elevated levels of thyroid-stimulating hormone (TSH)^7^. Levothyroxine replacement therapy is the first-line treatment for hypothyroidism^6,8^. Thyroid hormones influence the growth and homeostasis of gastrointestinal organs through the binding to receptors in the epithelium. They regulate some cellular processes such as proliferation, differentiation, apoptosis and metabolism, and some studies suggest their role in cancer progression^7,9,10^.

Several clinical studies have demonstrated that hypothyroidism could inhibit cancer cell proliferation, but data on the potential association between hypothyroidism and survival of PDAC patients are lacking^11,12^. The aim of this study was to examine the prevalence of hypothyroidism among patients with PDAC and assess its association with overall survival.

## Methods

Consecutive patients with any stage of PDAC according to the National Comprehensive Cancer Network (NCCN) classification^13^ between 2012 and 2021 at the University Hospitals of Parma, Modena and Pisa, were retrospectively reviewed using electronic medical records. The presence of hypothyroidism and treatment with levothyroxine was recorded. Cut-off values of FT4, FT3 and TSH were defined according to each laboratory protocol. Hypothyroidism was defined as low levels of FT4 (normal value 10–23 pmol/l). Normal values for TSH and FT3 were respectively, 0.4–4.0 mU/I and 5.4–12.3 pmol/l. Follow-up phone calls were used to assess the survival status of patients.

Patients aged 18 years or more with cytologically or histologically confirmed diagnosis of PDAC were included, while patients with a diagnosis of concomitant malignancy were excluded. The study protocol (344/2021/OSS/AOUPR, 8 July 2021) was approved by the local Ethics Committees, in accordance with the Declaration of Helsinki.

Continuous variables were reported as median and interquartile range (i.q.r.), and were compared using the Mann–Whitney *U* test. Categorical variables were reported as numbers and percentages, and were compared using chi-square or Fisher exact tests, as appropriate. Confidence intervals (c.i.) for prevalence estimates were obtained using the modified Wilson method. Overall survival (OS) was estimated using the Kaplan–Meier method.

Cox proportional hazards regression was used to assess the association between hypothyroidism and OS, after correcting for confounders. The following, prespecified confounders were adjusted in the Cox regression model: age, sex, T stage, N stage, adjuvant therapy use, log-transformed carbohydrate antigen 19-9(CA19-9) and log-transformed carcinoembryonic antigen (CEA). Missing covariate data were handled using multiple imputation with additive regression, predictive mean matching and bootstrapping under the missing at random assumption^14^. Resection margin was not imputed, as this variable was likely to be missing not at random.

Three sensitivity analyses were performed: Firth’s correction was used to assess sensitivity to sparse data bias; Royston–Parmar flexible parametric survival models were fitted instead of Cox regression models; missing data were handled with an alternative imputation procedure (that is, multivariate imputation by chained equations).

A *P* value <0.05 was considered statistically significant. All statistical analyses were performed in R, version 4.2.1 (R Foundation for Statistical Computing), as described in the *Supplementary material*. The authors have read the STROBE Statement—checklist of items, and the manuscript was prepared and revised according to the STROBE Statement—checklist of items. The study protocol was approved by the Ethics Committee (344/2021/OSS/AOUPR, 8 July 2021) which was in accordance with the Declaration of Helsinki. All patients signed an informed consent to authorize the scientific use of the collected data.

## Results

Baseline clinicopathological characteristics of the 493 patients included in this study are detailed in *Table 1*. In total, 75 patients had hypothyroidism. Patients with hypothyroidism were more frequently female (76 per cent (57/75) *versus* 44 per cent (184/418), *P* <0.0001) and were older (73 (68–79) years *versus* 71 (63–78) years; *P* = 0.045). The prevalence of hypothyroidism was 15.0 per cent (26/173) in locally advanced/metastatic patients and 15.3 per cent (49/320) in patients with resected PDAC (*P* = 0.78). Adjuvant therapy was more frequently used in the hypothyroidism group (23 *versus* 120, *P* = 0.14).

**Table 1:** Baseline demographics of the hypothyroidism and euthyroidism groups

Variables	Hypothyroidism (*n* = 75)	Euthyroidism (*n* = 418)	*P*
Age (years), median (i.q.r.)	73 (68–79)	71 (63–78)	0.045
**Sex**			<0.001
Female	57 (76)	184 (44)	
Male	18 (24)	234 (56)	
**Setting**			0.78
(Borderline) resectable	49 (65)	271 (65)	
Locally advanced	3 (4)	11 (3)	
Metastatic	23 (31)	136 (33)	
Adjuvant therapy after surgery	23/36 (64)	120/244 (49)	0.14
**Resection margin**			0.82
R0	19 (83)	84 (78)	
R1	4 (17)	23 (21)	
R2	0 (0)	1 (1)	
**T stage**			0.57
T1	1 (1)	20 (5)	
T2	22 (32)	105 (28)	
T3	32 (46)	171 (45)	
T4	14 (20)	84 (22)	
**N stage**			0.81
N0	15 (23)	96 (27)	
N1	33 (51)	169 (47)	
N2	17 (26)	94 (26)	
(CEA μg/l), median (i.q.r.)	6 (2–16)	5 (2–9)	0.33
CA19-9 (U/ml), median (i.q.r.)	233 (19–1001)	255 (34–1351)	0.39

Categorical variables are reported as *n* (%) unless indicated otherwise. CEA, carcinoembryonic antigen; i.q.r., interquartile range.

During the follow-up (median follow-up time, 11 months; i.q.r. 5–22 months), 48 patients with hypothyroidism died (70 per cent; 570 per 1000 person-years) *versus* 232 patients without hypothyroidism (63 per cent; 425 per 1000 person-years).

**Fig. 1:**
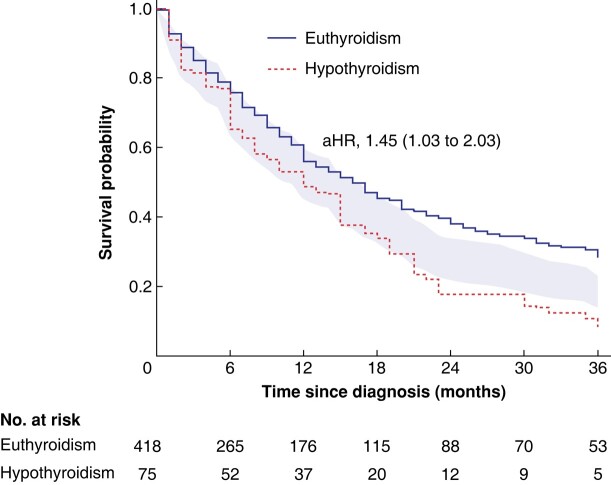
Covariate-adjusted Kaplan–Meier curve

Hypothyroidism was associated with worse OS, after adjusting for demographic and clinicopathological variables (HR 1.45; 95 per cent c.i. 1.03 to 2.03; *P* = 0.032) as shown in *Fig. 1* and *Table S1*. The adjusted difference in 1−, 2- and 3-year survival probability between patients with and without hypothyroidism was 8.0 per cent (−6.6 per cent to 22.6 per cent), 20.5 per cent (8.2 per cent to 32.7 per cent) and 20.2 per cent (10.8 per cent to 29.5 per cent) respectively. This association was similar between men and women (interaction test, *P* = 0.13), between resected PDAC and locally advanced/metastatic PDAC (interaction test, *P* = 0.59), and remained consistent in several sensitivity analyses (*Table S2*).

The grey shaded region indicates the ‘null zone’. If, at a certain timepoint, both Kaplan–Meier curves fall outside the null zone, then the difference in overall survival between the two curves at that timepoint is statistically significant. aHR, adjusted hazard ratio. The Kaplan–Meier curve and number at risk for the euthyroidism and hypothyroidism groups are shown in black and orange respectively.

## Discussion

Hypothyroidism might be associated with worse OS in PDAC patients regardless of sex and cancer stage; this association remained consistent across several sensitivity analyses. A relatively high prevalence of hypothyroidism was observed in PDAC patients, compared with the prevalence of hypothyroidism in the general population (15 per cent in PDAC patients *versus* 4–8 per cent in the general population)^15–17^. These findings might be explained by the increase in TSH levels associated with hypothyroidism. Recent preclinical studies showed that high TSH levels stimulated glioma proliferation and limited T cell killing^18^, and induced oxidative stress and genomic instability in mammary cells^19^.

The current study suggested an association between hypothyroidism and a higher mortality risk in PDAC patients. The higher mortality risk in hypothyroidism patients could potentially be associated with an increased risk of perineural invasion and nodal invasion in patients with hypothyroidism^20^, as Vascular Endothelial Growth Factor (VEGF) secretion is stimulated by TSH^21^; thus, TSH might promote metastasis by stimulating VEGF secretion and angiogenesis. Sarosiek *et al*. reported a 14 per cent prevalence of hypothyroidism in a retrospective analysis of 504 PDAC patients^22^, in line with the current findings.

The strengths of this study include a patient cohort of consecutive patients instead of a case-control design, correction for confounders that are important prognostic factors, a robust multiple imputation approach and consistent results across sensitivity analyses. Limitations include the retrospective nature of the study, a high prevalence of missing data for several prognostically important variables (for example, resection margin and use of neoadjuvant therapy), and the possibility of residual confounding due to baseline differences in clinicopathological variables and co-morbidities that were not determined in our study.

Further studies are needed to consider other factors such as neoadjuvant therapy use, performance status, co-morbidities and patient frailty. Additional studies should assess whether hypothyroidism is associated with disease-specific survival in PDAC. Furthermore, future studies should aim to develop optimal management strategies of patients with both PDAC and hypothyroidism.

## Supplementary Material

zrad119_Supplementary_Data

## Data Availability

Data supporting the findings of this study are available from the authors upon request.

## References

[1] Siegel RL, Miller KD, Wagle NS, Jemal A. Cancer statistics, 2023. CA Cancer J Clin 2023;73:17–48 36633525 10.3322/caac.21763

[2] Boyd LN, Ali M, Leeflang MM, Treglia G, de Vries R, Le Large TYS et al Diagnostic accuracy and added value of blood-based protein biomarkers for pancreatic cancer: a meta-analysis of aggregate and individual participant data. EClinicalMedicine 2023;55:101747 36457649 10.1016/j.eclinm.2022.101747 PMC9706531

[3] Kamisawa T, Wood LD, Itoi T, Takaori K. Pancreatic cancer. Lancet 2016;388:73–85 26830752 10.1016/S0140-6736(16)00141-0

[4] Balsano R, Tommasi C, Garajova I. State of the art for metastatic pancreatic cancer treatment: where are we now? Anticancer Res 2019;39:3405–3412 31262862 10.21873/anticanres.13484

[5] Digiacomo G, Volta F, Garajova I, Balsano R, Cavazzoni A. Biological hallmarks and new therapeutic approaches for the treatment of PDAC. Life 2021;11:843 34440587 10.3390/life11080843 PMC8400856

[6] Wu CC, Islam MM, Nguyen PA, Poly TN, Wang C-H, Iqbal U et al Risk of cancer in long-term levothyroxine users: retrospective population-based study. Cancer Sci 2021;112:2533–2541 33793038 10.1111/cas.14908 PMC8177794

[7] Krashin E, Piekiełko-Witkowska A, Ellis M, Ashur-Fabian O. Thyroid hormones and cancer: a comprehensive review of preclinical and clinical studies. Front Endocrinol 2019;10:59 10.3389/fendo.2019.00059 PMC6381772 30814976

[8] Pinto A, Glick M. Management of patients with thyroid disease: oral health considerations. J Am Dent Assoc 2002;133:849–858 12148678 10.14219/jada.archive.2002.0299

[9] Hall LC, Salazar EP, Kane SR, Liu N. Effects of thyroid hormones on human breast cancer cell proliferation. J Steroid Biochem Mol Biol 2008;109:57–66 18328691 10.1016/j.jsbmb.2007.12.008

[10] Lin H-Y, Tang H-Y, Shih A, Keating T, Cao G, Davis PJ et al Thyroid hormone is a MAPK-dependent growth factor for thyroid cancer cells and is anti-apoptotic. Steroids 2007;72:180–187 17174366 10.1016/j.steroids.2006.11.014

[11] Biondi B, Cooper DS. Thyroid hormone therapy for hypothyroidism. Endocrine 2019;66:18–26 31372822 10.1007/s12020-019-02023-7

[12] Rennert G, Rennert HS, Pinchev M, Gruber SB. A case-control study of levothyroxine and the risk of colorectal cancer. J Natl Cancer Inst 2010;102:568–572 20305129 10.1093/jnci/djq042 PMC2857798

[13] Tempero MA, Arnoletti JP, Behrman SW, Ben-Josef E, Benson AB, Casper ES et al Pancreatic adenocarcinoma, version 2.2012: featured updates to the NCCN guidelines. J Natl Compr Canc Netw 2012;10:703–713 22679115 10.6004/jnccn.2012.0073 PMC3807091

[14] Harrell J, Frank E. *Regression Modeling Strategies: With Applications to Linear Models, Logistic and Ordinal Regression, and Survival Analysis*. Cham: Springer, 2015

[15] Cappola AR, Fried LP, Arnold AM, Danese MD, Kuller LH, Burke GL et al Thyroid status, cardiovascular risk, and mortality in older adults. JAMA 2006;295:1033–1041 16507804 10.1001/jama.295.9.1033 PMC1387822

[16] Benseñor IM, Goulart AC, Lotufo PA, Menezes PR, Scazufca M. Prevalence of thyroid disorders among older people: results from the São Paulo Ageing & Health Study. Cad Saude Publica 2011;27:155–161 21340114 10.1590/s0102-311x2011000100016

[17] Díez JJ, Iglesias P. Malignant neoplasms in people with hypothyroidism in Spain: a population-based analysis. PLoS One 2022;17:e0275568 36197930 10.1371/journal.pone.0275568 PMC9534429

[18] Wu Z, Xi Z, Xiao Y, Zhao X, Li J, Feng N et al TSH-TSHR axis promotes tumor immune evasion. J Immunother Cancer 2022;10:e004049 35101946 10.1136/jitc-2021-004049 PMC8804696

[19] Peixoto MS, de Vasconcelos E Souza A, Andrade IS, de Carvalho el Giusbi C, Coelho Faria C, Hecht F et al Hypothyroidism induces oxidative stress and DNA damage in breast. Endocr Relat Cancer 2021;28:505–519 34010147 10.1530/ERC-21-0010

[20] Gasparini G, Pellegatta M, Crippa S, Lena MS, Belfiori G, Doglioni C et al Nerves and pancreatic cancer: new insights into a dangerous relationship. Cancers (Basel) 2019;11:893 31248001 10.3390/cancers11070893 PMC6678884

[21] Soh EY, Sobhi SA, Wong MG, Meng YG, Siperstein AE, Clark OH et al Thyroid-stimulating hormone promotes the secretion of vascular endothelial growth factor in thyroid cancer cell lines. Surgery 1996;120:944–947 8957478 10.1016/s0039-6060(96)80038-9

[22] Sarosiek K, Gandhi AV, Saxena S, Kang CY, Chipitsyna GI, Yeo CJ et al Hypothyroidism in pancreatic cancer: role of exogenous thyroid hormone in tumor invasion—preliminary observations. J Thyroid Res 2016;2016:2454989 27123358 10.1155/2016/2454989 PMC4830736

