# Supplementary material for: Association of hypothyroidism with survival in pancreatic cancer: retrospective cohort study
Source: BJS Open. 2024 Jan 9;8(1):zrad119. doi: 10.1093/bjsopen/zrad119 (PMC10776351; doi:10.1093/bjsopen/zrad119)
Supplement: zrad119_Supplementary_Data [file zrad119_supplementary_data.docx]

**Title**

**ASSOCIATION OF HYPOTHYROIDISM WITH SURVIVAL IN PANCREATIC CANCER: A RETROSPECTIVE COHORT STUDY**

Authors

Ingrid Garajová^1*^, Annalisa Comandatore^2*^, Lenka Boyd^3**^, Mahsoem Ali^3**^, Fabio Gelsomino^4^, Stefania de Lorenzo^5^, Giuseppe Pedrazzi^6^, Andrea Spallanzani^4^, Giulio Martinelli^4^, Rita Balsano^1^, Francesco Leonardi^1^, Matteo Palmeri^2^, Geert Kazemier^7^, Gregorio Di Franco^2^, Simone Guadagni^2^, Niccolò Furbetta^2^, Manuel Gentiluomo^8^, Niccolò Ramacciotti^2^, Giulio Di Candio^2^, Elisa Giovannetti^9^° and Luca Morelli^2^°

^1^ Medical Oncology Unit, University Hospital of Parma, 43126 Parma, Italy

^2^ General Surgery Unit, Department of Translational Research and New Technologies in Medicine and Surgery, University of Pisa, Pisa, Italy

^3^ Department of Medical Oncology, Lab of Medical Oncology, Cancer Center Amsterdam, Amsterdam UMC, VU University Medical Center (VUmc), De Boelelaan 1118, 1081 HZ

^4^ Department of Oncology and Hematology, University Hospital of Modena, Italy

^5^ Oncology Unit, Azienda USL Bologna, Bologna, Italy

^6^ Department of Medicine and Surgery, University of Parma, 43126 Parma, Italy

^7^ Department of Surgery, Amsterdam UMC, VU University Medical Center, De Boelelaan 1118, 1081 HZ

^8^ Department of Biology, University of Pisa, Pisa, Italy

^9^ Department of Medical Oncology, Lab of Medical Oncology, Cancer Center Amsterdam, Amsterdam UMC, VU University Medical Center (VUmc), De Boelelaan 1118, 1081 HZ, Postbus 7057, 1007 MB, Amsterdam, the Netherlands**;** Cancer Pharmacology Lab, AIRC Start-Up Unit, Fondazione Pisana per la Scienza, Via Ferruccio Giovannini, 13, 56017, San Giuliano Terme PI, Pisa, Italy

**Corresponding author: Luca Morelli, MD, FACS,** General Surgery Unit, Department of Translational Research and New Technologies in Medicine and Surgery, University of Pisa, Via Paradisa 2, Pisa 56124, Italy. [luca.morelli@unipi.it](mailto:luca.morelli@unipi.it) ORCID (0000-0002-7742-9556).

**Supplementary Materials - Index**

| **Supplementary Methods** |  |
| --- | --- |
| Statistical analysis | *pag. 3* |
| **Supplementary Tables** |  |
| Table S1. Association of hypothyroidism with overall survival in PDAC | *pag. 5* |
| Table S2. Sensitivity analyses | *pag. 6* |
| **References** | *pag. 7* |
|  |  |

**Supplementary Methods**

*Statistical analysis*

Continuous baseline variables were reported as median (interquartile range), and were compared between groups using the Mann-Whitney *U* test. Categorical variables were reported as numbers and percentages, and were compared between groups using chi-square or Fisher exact tests, as appropriate. Confidence intervals for prevalence estimates were obtained using the modified Wilson method. Overall survival was estimated using the Kaplan-Meier method.

Cox proportional hazards regression was used to assess the association between hypothyroidism and overall survival, after correcting for confounders. Confounders were selected based on subject matter knowledge and directed acyclic graphs, and data-based variable selection procedures were not performed. The following confounders were adjusted for in the Cox regression model: age, sex, T stage, N stage, adjuvant therapy use, log-transformed CA19-9, and log-transformed CEA. Resection margin was not imputed, as this variable was likely to be missing not at random. A likelihood ratio test was used to assess the statistical significance of the hypothyroidism variable. The functional form and proportional hazards assumption of each (continuous) variable was checked using Schoenfeld residuals and Martingale residuals, respectively. Nonlinearity of continuous variables was explored using restricted cubic splines with three knots at the 10^th^, 50^th^, and 90^th^ percentile.^1^ However, as there was no evidence for nonlinearity (nonlinearity test, P=0.45), the restricted cubic spline terms were removed from the final model. Collinearity was assessed using variance inflation factors.

Interaction terms were added to the Cox regression model to investigate whether the association between hypothyroidism and overall survival was different between men and women, and between locally advanced/metastatic PDAC and radically resected PDAC. This Cox regression model also included all previously mentioned confounders. Likelihood ratio tests were used to assess the statistical significance of the interaction terms.

Missing covariate data were handled using multiple imputation with additive regression, predictive mean matching, and bootstrapping.^1^ The imputation model was fully congenial with the Cox regression model, and included all potential covariates (age, sex, CEA, CA19-9, T stage, N stage, resection margin, use of adjuvant therapy, presence of hypothyroidism), as well as the event indicator (ie, whether the patient died or was censored) and the Nelson-Aalen estimator of the cumulative baseline hazard.^1,2^ This approach was used as multiple imputation using the event indicator and the cumulative baseline hazard is recommended for Cox regression models, and alternative methods (eg, using log-transformed time) can produce biased results.^2^ Continuous variables in this flexible imputation model were modelled using restricted cubic splines with three knots. In total, 60 imputation datasets and 100 bootstrap resamples were used.^1^

Overall survival was visualized using covariate-adjusted Kaplan-Meier curves, and was estimated using multiple imputation. For this purpose, inverse probability of treatment weighted Kaplan-Meier estimates were pooled across 60 imputed datasets that were generated using the multivariate imputation by chained equations method.^3-5^ In addition, the ‘null zone’ was calculated and visualized in the Kaplan-Meier curve as a grey shaded region. At any timepoint, the difference in overall survival is statistically significant if both curves are outside the null zone at that timepoint.^6^ The difference in 1 year, 2 year, and 3 year survival between patients with and without hypothyroidism was estimated using these covariate-adjusted Kaplan-Meier curves. Confidence intervals for the difference in survival probability were calculated using the method of Bland and Altman.^7^

Three sensitivity analyses were performed. First, Cox regression was performed with Firth’s correction (ie, penalized maximum partial likelihood estimation) to assess whether results were sensitive to sparse data bias.^8^ Second, Royston-Parmar flexible parametric survival models were fit to assess the impact of potential time-varying effects and the choice of scale (ie, the proportional hazards scale versus the proportional odds scale).^9^ In these models, the log cumulative hazard and the log cumulative odds were modeled using restricted cubic splines with three knots, as this number of knots optimized the Akaike Information Criterion. Third, multivariate imputation using chained equations was used instead of the flexible additive imputation model that was used in the main analyses.^4^ Single imputation was used for the first two sensitivity analyses.

A P value lower than 0.05 was considered to indicate statistical significance. All statistical analyses were performed in R, version 4.2.1 (R Foundation for Statistical Computing).

**Supplementary Tables**

**Table S1. Association of hypothyroidism with overall survival in PDAC.**

| **Variable** | **Hazard ratio**  **(95% confidence interval)** | | **P value** |
| --- | --- | --- | --- |
| Hypothyroidism | 1.45 | (1.03 to 2.03) | 0.032 |
| Age (per 10 years) | 1.13 | (1.00 to 1.29) | 0.034 |
| Female sex | 0.67 | (0.51 to 0.88) | 0.002 |
| Adjuvant therapy | 0.26 | (0.18 to 0.36) | <0.0001 |
| T stage |  |  | 0.0003 |
| T1 | - |  |  |
| T2 | 1.00 | (ref.) |  |
| T3 | 1.70 | (1.14 to 2.54) |  |
| T4 | 1.48 | (0.93 to 2.37) |  |
| N stage |  |  | 0.007 |
| N0 | 1.00 | (ref.) |  |
| N1 | 1.28 | (0.92 to 1.77) |  |
| N2 | 1.76 | (1.15 to 2.70) |  |
| Ln(CEA), μg/L | 1.13 | (1.01 to 1.26) | 0.024 |
| Ln(CA19-9), U/mL | 1.07 | (1.01 to 1.14) | 0.027 |
| P values are derived from likelihood ratio tests. T2 was chosen as the reference value, as there were only 6 patients with T1 stage in the cohort. Ref., reference value. | | | |

**Supplementary table 2. Sensitivity analyses.**

| **Variable** | **Hazard ratio**  **(95% confidence interval)** | | **P value** |
| --- | --- | --- | --- |
| Sensitivity analysis 1 | 1.51 | (1.09 to 2.07) | 0.015 |
| Sensitivity analysis 2 | 1.53 | (1.10 to 2.11) | * |
| Sensitivity analysis 3 | 1.43 | (1.03 to 1.97) | 0.036 |
| Sensitivity analysis 1: Firth’s bias reduction method  Sensitivity analysis 2: proportional hazards Royston-Parmar flexible parametric survival model (no P value provided)  Sensitivity analysis 3: multivariate imputation by chained equations instead of flexible additive imputation models to handle missing data. | | | |

**References**

1. Harrell J, Frank E. Regression modeling strategies: With applications to linear models, logistic and ordinal regression, and survival analysis; 2015.

2. White IR, Royston P. Imputing missing covariate values for the Cox model. *Statistics in medicine* 2009; **28**(15): 1982-98.

3. Xie J, Liu C. Adjusted Kaplan–Meier estimator and log‐rank test with inverse probability of treatment weighting for survival data. *Statistics in medicine* 2005; **24**(20): 3089-110.

4. Van Buuren S, Groothuis-Oudshoorn K. mice: Multivariate imputation by chained equations in R. *Journal of statistical software* 2011; **45**: 1-67.

5. Denz R, Klaaßen‐Mielke R, Timmesfeld N. A comparison of different methods to adjust survival curves for confounders. *Statistics in Medicine* 2022.

6. Boers M. Null bar and null zone are better than the error bar to compare group means in graphs. *Journal of clinical epidemiology* 2004; **57**(7): 712-5.

7. Altman DG, Bland JM. Interaction revisited: the difference between two estimates. *Bmj* 2003; **326**(7382): 219.

8. Greenland S, Mansournia MA, Altman DG. Sparse data bias: a problem hiding in plain sight. *bmj* 2016; **352**.

9. Royston P, Parmar MK. Flexible parametric proportional‐hazards and proportional‐odds models for censored survival data, with application to prognostic modelling and estimation of treatment effects. *Statistics in medicine* 2002; **21**(15): 2175-97.
